# The Palliative Performance Scale predicts mortality in hospitalized patients with COVID-19

**DOI:** 10.1177/0269216320940566

**Published:** 2020-07-17

**Authors:** Michele Fiorentino, Sri Ram Pentakota, Anne C Mosenthal, Nina E Glass

**Affiliations:** 1 Rutgers New Jersey Medical School, Newark, NJ, USA; 2 Tufts University School of Medicine, Boston, MA, USA

**Keywords:** Palliative care, coronavirus, frailty, hospital mortality, physical functional performance

## Abstract

**Background:**

Coronavirus disease 2019 (COVID-19) has a substantial mortality risk with increased rates in the elderly. We hypothesized that age is not sufficient, and that frailty measured by preadmission Palliative Performance Scale would be a predictor of outcomes. Improved ability to identify high-risk patients will improve clinicians’ ability to provide appropriate palliative care, including engaging in shared decision-making about life-sustaining therapies.

**Aim:**

To evaluate whether preadmission Palliative Performance Scale predicts mortality in hospitalized patients with COVID-19.

**Design:**

Retrospective observational cohort study of patients admitted with COVID-19. Palliative Performance Scale was calculated from the chart. Using logistic regression, Palliative Performance Scale was assessed as a predictor of mortality controlling for demographics, comorbidities, palliative care measures and socioeconomic status.

**Setting/participants:**

Patients older than 18 years of age admitted with COVID-19 to a single urban public hospital in New Jersey, USA.

**Results:**

Of 443 admitted patients, we determined the Palliative Performance Scale score for 374. Overall mortality was 31% and 81% in intubated patients. In all, 36% (134) of patients had a low Palliative Performance Scale score. Compared with patients with a high score, patients with a low score were more likely to die, have do not intubate orders and be discharged to a facility. Palliative Performance Scale independently predicts mortality (odds ratio 2.89; 95% confidence interval 1.42–5.85).

**Conclusions:**

Preadmission Palliative Performance Scale independently predicts mortality in patients hospitalized with COVID-19. Improved predictors of mortality can help clinicians caring for patients with COVID-19 to discuss prognosis and provide appropriate palliative care including decisions about life-sustaining therapy.

**What is already known about the topic?**

- Mortality rates in COVID-19 range from 0.4% to 16.3% with increased rates in hospitalized patients, the elderly and other vulnerable populations.
- Studies in other seriously ill individuals demonstrate that frailty is a predictor of mortality.

**What this paper adds?**

- Frailty, measured by the preadmission Palliative Performance Scale, is independently predictive of mortality in patients admitted with COVID-19.

**Implications for practice, theory or policy**

- Incorporating the Palliative Performance Scale into assessment of patients with COVID-19 can help predict outcomes.
- Improved understanding of mortality risk can help clinicians caring for patients with COVID-19 to discuss prognosis and provide appropriate palliative care including appropriate recommendations about life-sustaining therapy.

## Introduction

On 11 March 2020, The World Health Organization declared coronavirus disease 2019 (COVID-19) a global pandemic and as of 23 May, 5 million cases had been confirmed.^1^ Overall mortality of COVID-19 ranges from 0.4% to 16.3%^2^ with increased rates in hospitalized patients, the elderly, and other vulnerable groups.^3–5^ Although advanced age is clearly a predictor of poor outcomes in COVID-19,^6^ data to help accurately predict risk are still lacking.

In other conditions, frailty has been found to be a predictor of mortality,^7^ and its evaluation in COVID-19 has been recommended.^8^ However, there has been a paucity of literature evaluating the effect of frailty in patients with COVID-19. Improved ability to identify patients at high risk of death, will improve clinicians’ ability to provide appropriate palliative care, including engaging in shared decision-making with our patients about life-sustaining therapies. Many frailty assessments exist. However, many are complicated and hard to determine at the bedside in critically ill patients where history is limited, and patients may not be able to participate physically or even verbally in the assessment. The Palliative Performance Scale, on the other hand, consists of only five domains, which allows it to be easily calculated at the bedside based on history from patients or their families. The Palliative Performance Scale is a validated tool to assess frailty and to prognosticate survival in seriously ill populations.^9,10^

We sought to determine whether a frailty measure, such as this would correlate with mortality. We, therefore, applied the Palliative Performance Scale to patients hospitalized during the initial COVID-19 surge in a public urban hospital. We hypothesized that a low preadmission Palliative Performance Scale score would independently predict mortality in hospitalized patients with COVID-19.

## Methods

### Data source and study population

We performed a retrospective observational cohort study of all patients with a positive COVID-19 RNA nasopharyngeal swab admitted to an urban public hospital that treats a largely underserved population in Newark, New Jersey from 15 March to 10 April 2020. Study staff abstracted demographic data (age, sex, race/ethnicity, admission source, and insurance status), clinical data (body mass index, Charlson comorbidity index,^11^ and preadmission Palliative Performance Scale score) and details of the hospital course (intensive care unit admission, intubation, haemodialysis, discharge disposition and length of stay) from the electronic medical record. The preadmission Palliative Performance Scale was calculated using information available in the medical chart about the patient’s performance status prior to admission and contracting COVID-19. Using this information, the score was calculated by a physician member of the study team. To further investigate palliative care processes and interventions, we reviewed the charts for do not resuscitate, do not intubate and comfort measures only orders.

The Rutgers New Jersey Medical School Institutional Review Board approved this study. This study was granted a waiver of consent and a waiver of Health Insurance Portability and Accountability Act authorization, since it is a retrospective study that involves no more than minimal risk to subjects (Reference number Pro2019000864; Approved 6 April 2020).

### Outcomes

The primary outcome of this study was in-hospital mortality.

### Palliative Performance Scale

Anderson et al.,^12^ developed the Palliative Performance Scale to help assess prognosis in cancer patients receiving palliative care and it has since been applied to other seriously ill populations.^9,13–15^ The score is calculated from five domains: ambulation, activity and evidence of disease, self-care, intake and level of consciousness. Scores range from 0 to 100. Prior studies in seriously ill individuals have used the Palliative Performance Scale as a measure of frailty. In these studies, a score ⩽70 was predictive of in-hospital mortality and poor functional outcome at discharge, therefore, we dichotomized Palliative Performance Scale scores as low (⩽70) and high (>70).^12^

Palliative Performance Scale scores are easy to determine from interviewing patients or families and can be estimated by reviewing the medical records; patients whose charts did not include sufficient information to calculate the Palliative Performance Scale were excluded.

### Statistical analysis

To evaluate for selection bias in our enrolled patients, we first compared patients with Palliative Performance Scale and without Palliative Performance Scale scores (excluded from the study). We found no statistically significant differences on most patient, clinical and outcome variables; except for the group without Palliative Performance Scale scores had shorter length of stay. Among the study patients (with Palliative Performance Scale scores), we first performed descriptive analyses, using counts and proportions for categorical variables, means and standard deviations for normally distributed continuous variables (age and body mass index), and medians and first and third quartiles for skewed continuous variables (length of hospital stay, length of intensive care unit stay and days on ventilator) for the entire cohort and by low and high Palliative Performance Scale groups. Second, low and high Palliative Performance Scale groups were compared using chi-square test or Fischer’s exact test for categorical variables and Student’s t-test and the Mann–Whitney U test for continuous variables, which were not and were skewed, respectively. We fit series of logistic regression models beginning with unadjusted model to assess association between Palliative Performance Scale and in-hospital mortality. Adjusted odds ratios for in-hospital mortality were obtained from sequentially fit multivariable logistic regression models by adding the following covariates at each stage: age categories, gender, race/ethnicity; body mass index and Charlson comorbidity index; do not intubate orders; dialysis and insurance. A *p*-value of 0.05 or less was a priori determined as cut-off value to be used to infer statistically significant associations. All analyses were performed using SAS v9.4 (SAS Institute, Cary NC).

## Results

Of 443 patients admitted with COVID-19 during the study period, 374 were eligible for inclusion after excluding 61 patients for inability to calculate their Palliative Performance Scale and eight patients who remained in the hospital at the conclusion of the study.

Thirty-six percent of patients had low a Palliative Performance Scale (134/374). The low Palliative Performance Scale group was older, predominantly black (78%) and had more comorbidities. High Palliative Performance Scale patients were admitted largely from home (>90%), whereas only 50% of patients with a low score were admitted from home, with most others being transferred from another healthcare facility (Table 1). Rates of intensive care unit admission and intubations were similar between the two groups (Table 2). A greater percentage of low Palliative Performance Scale patients had do not resuscitate and do not intubate orders placed during their hospitalization. The palliative care team was involved in the care of 28% (95% confidence interval 24%–33%) of all patients and 61% of patients that ultimately died.

**Table 1.** Patient characteristics by low and high Palliative Performance Scale groups (*n* = 374).

*n* **(%)**	**Low** PPS *n* = 134	High PPS *n* = 240	*p*-value
Age			
<40	4 (3%)	32 (13%)	<0.0001
40−49	5 (4%)	42 (18%)	
50−59	17(13%)	69 (29%)	
60−69	44 (33%)	59 (25%)	
70−79	30 (22%)	28 (12%)	
>80	34 (25%)	10 (4%)	
Mean Age ± *SD*	**69** **±** **13**	**56** **±** **14**	**<0.0001**
Gender			
Male	70 (52%)	149 (62%)	0.064
Race/ethnicity			
Black	104 (78%)	121 (50%)	<0.0001
White	7 (5%)	9 (4%)	
Hispanic	13 (10 %)	81 (34%)	
Other	10 (7%)	29 (12%)	
Charlson comorbidity index count			
Zero	1(1%)	51 (21%)	<0.0001
One	4 (3%)	58 (24%)	
Two	12 (9%)	50 (21%)	
Three	21 (16%)	37 (15%)	
Four	17 (13%)	24 (10%)	
>Four	79 (59%)	20 (8%)	
Charlson comorbidity index median (IQR)	5 (3, 6)	2 (1, 3)	<0.0001
Body mass index classifications			
Underweight	4 (3%)	3 (1%)	0.0011
Normal weight	38 (28%)	32 (13%)	
Overweight	41 (31%)	83 (35%)	
Obese	49 (37%)	122 (51%)	
Mean BMI ± *SD*	27 ± 8	30 ± 8	<0.0001
Insurance status			
Private	20 (15%)	81 (34%)	<0.0001
Medicare	76 (57%)	43 (18%)	
Medicaid	28 (21%)	36 (15%)	
Charity care	1 (1%)	9 (4%)	
None	9 (7%)	61 (25%)	
Other	0 (0%)	10 (4%)	
Admit from			
Home	67 (50%)	219 (91%)	<0.0001
Homeless/shelter	2 (1%)	15 (6%)	
Skilled nursing facility/long-term care facility	56 (42%)	1 (0%)	
Acute rehab facility	2 (1%)	0 (0%)	
Prison	0 (0%)	4 (2%)	
Group home	6 (4%)	1 (0%)	
Long-term acute care facility	1 (1%)	0 (0%)	

PPS: Palliative Performance Scale; IQR: interquartile range.

**Table 2.** Hospital outcomes and other significant factors stratified by low and high Palliative Performance Scale.

*n* **(%)**	Low PPS *n* = 134	High PPS *n* = 240	*p*-value
ICU admission	33 (25%)	82 (34%)	0.055
Intubation	32 (24%)	56 (23%)	0.905
New onset dialysis	8 (6 %)	21 (9%)	<0.0001
Do not intubate order	47 (35%)	21 (9%)	<0.0001
Do not resuscitate order	77 (57%)	53 (22%)	<0.0001
Comfort measures only	45 (34%)	20 (8%)	<0.0001
Length of stay median (IQR)	6 (3, 10)	7 (4, 13)	0.034
Discharge disposition			
Home	36 (27%)	156 (65%)	<0.0001
Skilled nursing facility/long-term care facility	24 (18%)	7 (3 %)	
Acute rehab facility	1 (1%)	10 (4%)	
Prison	0 (0%)	4 (2%)	
Hospice	9 (7%)	0 (0%)	
Long-term acute care facility	1 (1%)	0 (0%)	
Psychiatric admission	0 (0%)	2 (1%)	
Transfer	0 (0%)	4 (2%)	
Field hospital	0 (0%)	2 (1%)	
Death	63 (47%)	55 (23%)	

PPS: Palliative Performance Scale; IQR: interquartile range; ICU: intensive care unit.

In-hospital deaths were more common in the low Palliative Performance Scale group (47% (95% confidence interval 39–56%) versus 23% (95% confidence interval 18–28%)). Most (81% of) intubated patients died. Only 1 (3%) of 32 low Palliative Performance Scale patients survived intubation, compared with 29% of patients with a high score. Over half (59%) of low Palliative Performance Scale patients who were intubated were subsequently made comfort measures only compared with 25% among high-score patients.

Multivariable logistic regression analyses showed that with a low preadmission Palliative Performance Scale, the odds of dying in the hospital were 2.89 (95% confidence interval 1.42–5.85) times higher than with a high score (Table 3). This association persisted when adjusting for COVID-19-specific treatments.

**Table 3.** Multivariable logistic regression for association between Palliative Performance Scale and in-hospital mortality (*n* = 374).

	Odds ratio (95% CI)
PPS	
Low (⩽70)	**2.89 (1.42−5.85)**
High (>70)	Reference
Age	
<40	Reference
40−49	0.80 (0.17–3.72)
50−59	1.86 (0.30–11.67)
60−69	2.14 (0.31–14.61)
70−79	5.97 (0.78–45.62)
⩾80	5.45 (0.66–45.03)
Race/ethnicity	
Non-Hispanic Black	Reference
Hispanic	1.53 (0.70–3.35)
Non-Hispanic White	2.23 (0.67–7.39)
Other race–non Hispanic	**3.78 (1.56–9.16)**
Gender	
Male	0.78 (0.44–1.38)
Body mass index classification	
Normal weight	Reference
Obese	0.78 (0.37–1.65)
Overweight	0.84 (0.39–1.79)
Underweight	0.36 (0.05–2.41)
Charlson comorbidity index	
Zero	Reference
One	0.46 (0.08–2.60)
Two	1.15 (0.17–7.68)
Three	0.87 (0.13–5.98)
Four	1.01 (0.13–7.63)
>Four	0.81 (0.10–6.34)
Do not intubate order (no)	**3.66 (1.65–8.10)**
Dialysis	
None	Reference
New onset dialysis	**24.72 (7.98–76.64)**
Previously dialysis dependent	0.99 (0.21–4.64)
Insurance status	
Private	Reference
Charity care	0.72 (0.09–5.90)
Medicaid	1.25 (0.49–3.16)
Medicare	0.56 (0.25–1.24)
None	1.16 (0.45–2.97)
Other	2.57 (0.41–16.04)

PPS: Palliative Performance Scale; CI: confidence interval.

## Conclusion

### Main findings

Among hospitalized patients with COVID-19, mortality was 31% overall, and significantly higher (81%) in intubated patients. Frailty, assessed by low Preadmission Palliative Performance Scale, independently predicted mortality in hospitalized patients. Surprisingly, age and Charlson comorbidity index did not independently predict mortality.

### What this study adds?

The Palliative Performance Scale is a tool that can be easily administered at the bedside on presentation. With the known high rates of mortality, especially in the elderly^3^ and those coming from nursing homes with COVID-19,^4^ bedside providers frequently had conversations about end of life care and patients elected to be do not intubate early in their hospitalization. Previously, we have used the Palliative Performance Scale to flag patients with high mortality risk for palliative care consultation. However, this study establishes that the Palliative Performance Scale can also be used to help intensive care unit clinicians’ ability to prognosticate. During the COVID-19 pandemic, many hospitals in the region, including ours, were operating over capacity with additional makeshift intensive care units set up to care for critically ill patients. Expanded palliative care services were available during the surge response in our hospital. However, most initial goals of care conversations including conversations about withholding life-sustaining treatment occurred with bedside providers. During this time, the palliative care team primarily provided ongoing support for families. Although most patients with a do not intubate order died, the do not intubate order was likely not the cause of their death especially when considering the high rate of mortality in intubated patients. Of note, in other patient populations, early use of palliative care with seriously ill patients, does not increase mortality.^16^ Recognizing frailty as a predictor of mortality will help guide conversations and inform recommendations for appropriate palliative care processes including life-sustaining therapy.

This short report confirms that low Palliative Performance Scale, a marker of frailty, independently predicts death in COVID-19. This finding is not surprising as many studies have found poor outcomes in the elderly,^5^ especially those with comorbidities which both contribute to frailty. Following these initial reports, published guidelines advocate the evaluation of frailty in caring for elderly patients^8,17^ yet to our knowledge, there are limited studies evaluating frailty as an independent predictor of outcomes in COVID-19.

Although there are a multitude of frailty indexes, many are overly complex and multivariable. This information can be hard to obtain from patients when they are critically ill or inaccurate when relying on families, especially during a pandemic. For example, the frailty index that was used by Bellelli et al.^18^ to predict in-hospital mortality in hospitalized patients during COVID-19 includes 43 variables. Although these findings were similar to ours, we argue that the use of such a complex scale during a pandemic is time-consuming and impractical especially with the no visitor policy that has been set at many hospitals in the United States. The time it would take to complete this scale would result in delays in obtaining an assessment and decrease the availability to use frailty as a tool in guiding conversations. The Palliative Performance scale only consists of five domains and can easily be calculated at the bedside in minutes. With early access to the score, clinicians can use these findings to discuss prognosis and guide goals of care conversations.

### Limitations of the study

Limitations to this report include that it is a single centre study at an institution that has been heavily affected by COVID-19. However, the degree of our surge heightened our ability to identify patients during a short time period and to observe the correlation between preadmission Palliative Performance Scale and outcomes. In this retrospective study, Palliative Performance Scale was abstracted from the chart and was dependent on accurate documentation. A more accurate assessment could be obtained from direct patient interview. Moreover, the retrospectively calculated scores could have been biased by knowing the outcome, in-hospital mortality, at the time of retrospectively calculating them. It is, therefore, a major limitation that the scores were not calculated at admission, rather were calculated retrospectively. However, we hypothesize that any bias introduced would be towards a higher score based on data supporting patient’s ability to meet each of the thresholds for score (walking, activities of daily living, etc.), therefore, strengthening our findings. Furthermore, in dichotomizing the variable, there would be less opportunity for error.

## Conclusion

In conclusion, patients admitted with COVID-19 and low preadmission Palliative Performance Scale are nearly three times more likely to die in the hospital and survivors are more likely to be discharged to a facility. Incorporating the Palliative Performance Scale into the initial assessment of all patients with COVID-19 could help predict outcomes. Moreover, improved predictors of mortality will help clinicians caring for patients with COVID-19 to discuss prognosis and make informed decisions about life-sustaining therapy.
